# Preparation and Characterization of Poly(lactic acid) Membranes and Films Coated with Polyaniline for Potential Use in Environmental Remediation

**DOI:** 10.1021/acsomega.3c06659

**Published:** 2024-01-17

**Authors:** Ana Daymi Cabrera Gonzalez, José Ramón Flores León, Claudia Georgina Ramirez Mendoza, Dora Evelia Rodríguez Félix, María Mónica Castillo Ortega, Hisila Santacruz Ortega, Francisco Rodríguez Félix, Tomás Jesús Madera Santana, Jesús Manuel Quiroz Castillo

**Affiliations:** † Departamento de Investigación en Polímeros y Materiales, Universidad de Sonora, Hermosillo C.P. 83000, Sonora, Mexico; ‡ Departamento de Investigación y Posgrado en Alimentos, Universidad de Sonora, Hermosillo C.P. 83000, Sonora, Mexico; § Laboratorio de Envases, CTAOV, Centro de Investigación en Alimentos y Desarrollo A.C., Hermosillo C.P. 83304, Sonora, Mexico

## Abstract

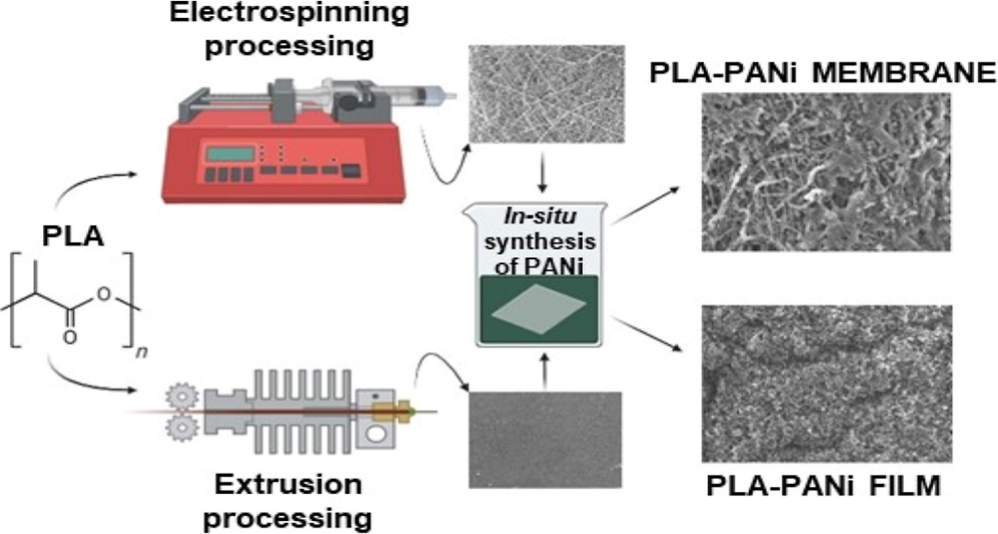

This research outlines the fabrication of polymeric membranes and films of poly(lactic acid) (PLA), prepared *via* electrospinning and extrusion, respectively. These materials were subsequently coated with polyaniline (PANi) by using the *in situ* chemical polymerization technique. Scanning electron microscopy micrographs revealed that the best coatings were achieved when 3 and 30 min of contact time with the monomeric solution were used for the membrane and film, respectively. Additionally, Fourier transform infrared spectra, thermogravimetric studies, and contact angle measurements demonstrated proper interaction between PLA and PANi. The findings of these studies suggest that PLA membranes and films can serve as suitable substrates for the deposition of PANi, and the composite materials hold potential for use in environmental remediation applications.

## 1 Introduction

A current global problem is the availability of drinking water. This situation has worsened recently due to the presence of anthropogenic contaminants from different productive sectors. Around the world, around two million tons of sewage are discharged into bodies of water daily.^1^ Studies from the United Nations Organization report a global average annual generation of 1500 km^3^ of wastewater, and this value is around 6 times greater than the water present in rivers around the world;^2^ in addition, it is reported that 90% of the water sources are contaminated, and this is mainly because 70% of domestic drainage and 33% of industrial drainage are discharged directly into rivers and lakes, without receiving any type of prior treatment.^3^ Also in recent years, an increase in the concentration of so-called emerging pollutants in wastewater has been observed. These substances are compounds that have little or no environmental regulation, even in developed countries, and that can also pose a risk to human health.^4^ Some examples of emerging contaminants are drugs, personal care products, food preservatives, plasticizers, disinfectants, hormones, and pesticides.^5^

In accordance with our living standards and to satisfy the need for food of the growing human population, in the industrial and agricultural sectors, the production and use of agrochemicals, mainly pesticides, has gradually increased, causing the contamination of surface and groundwater.^6^ Pesticides are nothing more than substances or a mixture of substances used for the prevention, elimination, or control of any type of pest capable of attacking crops or reducing the presence of insects. These are classified according to the type of pests they control as insecticides, fungicides, acaricides, nematicides, rodenticides, molluscicides, ovicides, and herbicides, the latter being one of the largest environmental pollutants worldwide, because they are used in most crops for weed control.^7^ After said application, a small part of these herbicides can protect agricultural products, while a high percentage of these are dispersed in the environment through hydrolysis, volatilization, or microbial action, causing a harmful effect because of their good solubility and mobility in aqueous media, their relatively low biodegradability, and their low persistence in soil.^8−11^ These substances have been considered by many researchers to be highly toxic to humans and marine organisms, causing severe cardiovascular problems, nervous system disorders, hormonal disorders, muscle degeneration, cancer, and genotoxicity.^12−15^ Therefore, it is of vital importance to remove these emerging pollutants from the environmental system, as it is very harmful to ecosystems.

For the efficient removal of herbicides in aqueous solutions, various physical, chemical, and biological processes have been developed. Among them, the adsorption of these emerging pollutants is the most effective technique, due to its low cost and high elimination efficiency, in addition, it constitutes a simple process and causes the generation of fewer byproducts.^16^ In this sense, different adsorbent materials have been used, for example, composite materials with clays,^17^ magnetic nanocomposites,^7^ activated carbon,^18^ metal–organic structures (MOF),^16^ polymer inclusion membranes,^13^ and polymer matrices.^19^

The polymeric matrices used for adsorption are light and chemically stable, have a high affinity for organic contaminants, and can be regenerated for reuse.^20^ Recent studies reported the use of compounds of sugar cane bagasse and sugar cane bagasse with polyaniline (PANi), polypyrrole, and sodium alginate for the adsorption of 2,4-dichlorophenoxyacetic acid. The adsorbent materials produced presented a maximum adsorption capacity of 17.75, 16.85, 21.01, and 7.6 (mg/g), respectively; the adsorption isotherms behaved according to the Freundlich model, and the adsorption kinetics can be described according to the pseudo-second-order model.^21^ Other investigators formulated an adsorbent material combining PANi, polypyrrole, and silicon dioxide for the effective adsorption of 2,4-dichlorophenol. The material obtained a removal capacity of 24.90 mg/g, and the adsorption process was better described according to the Langmuir isotherm model, in addition, it showed a very good reusability, which constitutes an excellent advantage in the adsorption process.^19^

PANi is a polymer that presents very favorable properties for the adsorption of organic compounds, such as environmental stability and ease of preparation;^22^ however, it presents the disadvantage of having poor mechanical properties such as low resistance and high brittleness, which is due to its highly aromatic nature that favors π–π interactions and interchain hydrogen bonding. A possible solution to this problem is the development of a composite material, where the PANi phase acts as an active component, and a matrix of another polymer is the one that provides the desired mechanical properties. The preparation of this type of material includes the synthesis of PANi on a polymeric matrix of the material with the appropriate mechanical properties.^23^

Poly(lactic acid) (PLA) belongs to the family of aliphatic esters commonly derived from α-hydroxy acids, which include poly(glycolic acid) and poly(mandelic acid). It is a thermoplastic polymer that has garnered significant attention due to its outstanding mechanical properties, such as high tensile strength and a high Young’s modulus. PLA can be produced from renewable natural materials and can be easily processed using standard plastic molding equipment to fabricate components, films, or fibers.^24−26^ Given these attributes, several researchers employed this polymer as a matrix in composite materials. In a study conducted by Yang *et al.* (2015), films based on PLA and lignin nanoparticles were prepared using two processing techniques: melt extrusion and solvent casting. The results indicated a notable enhancement in the mechanical properties of films obtained through extrusion, attributed to the effective dispersion of nanoparticles within the PLA matrix, promoting their interaction.^27^ In another study by Qian *et al.* (2018), biofilms of PLA were produced with the inclusion of bamboo cellulose as a nanofiller. The resulting material, with a specific nanofiller content, exhibited a relatively high level of transparency and tensile modulus.^28^ Furthermore, Arrieta *et al.* (2015) developed flexible materials from PLA and poly(hydroxyalkanoate) using the electrospinning technique, demonstrating improvements in tensile strength and a high elongation capacity.^29^

Taking into consideration the aforementioned facts, this research delineates the preparation and characterization of electrospun membranes and extruded films of PLA coated with PANi through an *in situ* chemical polymerization technique. To the best of our knowledge, this constitutes the first instance of manufacturing composite PLA–PANi films *via* the extrusion molding technique. This approach holds the promise of yielding materials with outstanding mechanical properties, representing a significant advancement in the field of composite materials.^27^ In the case of composite electrospun membranes, this study aims to design materials that exhibit a high ratio of surface area to volume or mass. These electrospun membranes provide an excellent opportunity for surface functionalization and present distinct mechanical properties, making them an appealing choice for various applications.^30^ Considering the combination of all of these attributes, both materials could potentially serve as promising candidates for deployment as adsorbents in the removal of herbicides from aqueous solutions.

## 2 Materials and Methods

### 2.1 Materials and Reagents

Hydrochloric acid (HCl, 37.2% purity), ammonium persulfate (APS, 98% purity), aniline (ANi, 99.5% purity), and 2,2,2-trifluoroethanol (TFE, 99% purity) were procured from Sigma-Aldrich. PLA (MW = 145,000 g/mol) was sourced from NatureWorks.

### 2.2 Preparation of PLA Membranes Using the Electrospinning Technique

For the preparation of the PLA membranes, the following procedure was followed: initially, a 10 mL solution was prepared by dissolving 1.4 g of PLA in TFE until the total volume reached 10 mL. The resulting solution was then subjected to magnetic stirring at room temperature for 24 h. In the production of the PLA nanofibers, certain parameters were adjusted based on those previously reported in the literature.^31^ A 14% PLA solution was transferred to a 5 mL plastic syringe, which was connected to a KDS Scientific peristaltic pump. The solution was dispensed at a rate of 2 mL/h, and a voltage of 15 kV was applied by using a Spellman model CZE 1000R power source. The distance between the needle tip and collector was fixed at 15 cm. The resulting fibers were collected on a square aluminum plate measuring 10 × 10 cm.

### 2.3 Preparation of PLA Films

In a 250 mL beaker, PLA pellets were introduced. These pellets underwent processing through the extrusion molding method employing an Atlas extruder LME model. The processing parameters were set as follows: a rotor speed of 31 rpm, a roller speed of 2.8 rpm, an extruder barrel temperature of 160 °C, and a die temperature of 170 °C. To maintain the desired flat geometry of the resulting film, an air cooling system was utilized at the extruder outlet. Upon successful processing, the film was sectioned and hermetically stored in a sealed container for further analysis and experimentation.

### 2.4 Preparation of Pure PANi and PLA–PANi Composite *via In Situ* Chemical Polymerization

For the synthesis of PANi, a specific procedure was followed. Initially, the aniline monomer underwent vacuum distillation for purification. Subsequently, a 0.5 M aniline solution was prepared by dissolving aniline in an aqueous solution of hydrochloric acid (HCl) with a concentration of 0.02 M. This solution was vigorously stirred for 20 min. Following this stage, 10 mL of the freshly prepared solution was transferred to a precipitation vessel. Subsequently, 10 mL of an acidic solution of ammonium persulfate [(NH_4_)_2_S_2_O_8_] with a concentration of 0.5 M was added dropwise, and the mixture was agitated for 3 h. It is worth noting that this synthesis was conducted in an ice bath to maintain the temperature at approximately 5 °C. Upon completion of the agitation process, a greenish-black precipitate formed, which was then filtered and thoroughly washed with Milli-Q water. Finally, the resulting solid was dried at 60 °C for 24 h.^32^

For the coating of polymeric membranes and films with PANi, initially, the aniline monomer underwent a vacuum distillation process to ensure its quality. Subsequently, a 0.5 M solution of aniline was prepared by dissolving aniline in an aqueous solution of hydrochloric acid (HCl) with a concentration of 0.02 M. Following this, polymeric membranes and films were immersed in the aniline solution for varying time periods. For the membranes, immersion times were set at 3, 5, and 15 min, while for the films, immersion times of 5, 15, 30, and 60 min were employed. This stage allowed the aniline to impregnate the membranes and films, preparing them for the subsequent phase. Subsequently, the materials treated with aniline were introduced into an aqueous solution of ammonium persulfate [(NH_4_)_2_S_2_O_8_] with a concentration of 0.5 M. This solution acted as an oxidizing agent to facilitate the *in situ* polymerization of aniline. For the membranes, the exposure time to this solution was 10 min, whereas for the films, a period of 20 min was used. This step was crucial in inducing the polymerization of aniline on the surfaces of the membranes and films. Finally, the coated materials were allowed to air-dry at room temperature for a period of 24 h. This process ensured that the materials were completely dry and ready for subsequent characterization and evaluation.^32^

### 2.5 Scanning Electron Microscopy

Following the acquisition of the pure membranes and composite polymeric films, morphological characterization was conducted using a JEOL 5410LV scanning electron microscope. For this examination, the samples were coated with a thin layer of gold, and a high-vacuum environment was maintained while utilizing an electron beam with an intensity of 20 kV. Moreover, the morphology of the cross sections of the samples was examined, which were obtained by sectioning the films and polymeric membranes in liquid nitrogen. Additionally, the diameter of the electrospun fibers was quantified using ImageJ software with measurements taken on a minimum of 54 individual fibers.

### 2.6 Fourier Transform Infrared Spectroscopy

The Fourier transform infrared (FTIR) spectra of PANi, polymeric membranes, films, and composite materials were acquired by using a PerkinElmer Frontier model FTIR spectrometer. The attenuated total reflectance technique was employed for the processed materials, while the KBr technique was used for PANi. This investigation aimed to elucidate the spectra of the individual components as well as the composite materials, with the objective of identifying the presence or absence of distinctive functional groups and potential interactions among them.

### 2.7 Thermogravimetric Analysis

Thermogravimetric analysis (TGA) of PANi, pure materials, and composites was performed by using a PerkinElmer Pyris 1 TGA instrument. Approximately 3 mg of the samples was carefully weighed into porcelain sample holders. The samples were then subjected to gradual heating, ranging from 25 to 600 °C, with a heating rate of 10 °C/min in an air atmosphere. To determine the thermal degradation temperatures, software associated with the equipment was employed to generate the derivative thermogravimetric analysis (DTG) curves from the thermograms.

### 2.8 Contact Angle

The contact angle with water for the pure and composite polymer membranes and films was determined by using the sessile drop method. Deionized water droplets were carefully placed on the surfaces of the membranes and polymeric films. This measurement was conducted ten times at various locations on each membrane and film surface. The results were reported as the mean ± the standard deviation.

### 2.9 Tensile Strength Tests

The mechanical properties of both the pure and composite polymeric films were assessed by using a United SSTM-5kN universal testing machine, which was equipped with a 5 kN load cell. The testing was conducted at a constant crosshead speed of 1 mm/min with a jaw spacing of 22.5 mm. The film thickness was measured using a Mitutoyo micrometer, and the dimensions were maintained within a range of 0.3–0.7 mm in thickness and a width of 5.0 mm. Micromechanical tests were conducted on the obtained PLA membranes, utilizing an ElectroForce 5110 apparatus and WinTest 7 software. The fibers were cut into striplike shapes measuring 5 mm in length by 2 mm in width. These test specimens were positioned at a distance of 12 mm and stretched at a rate of 1 mm/s until they reached the point of failure.

## 3 Results and Discussion

### 3.1 Scanning Electron Microscopy

**Figure 1:**
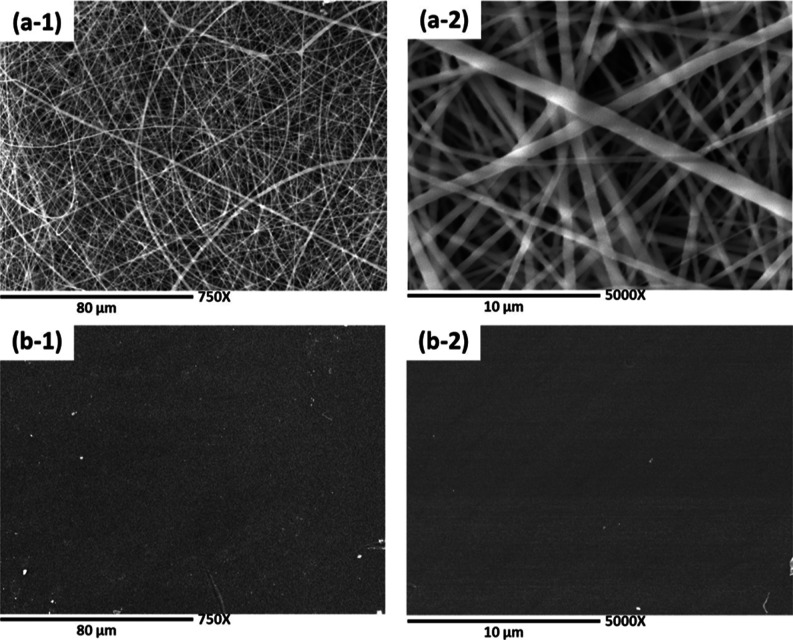
SEM micrographs of the (a-1) pure PLA membrane at 750× magnification, (a-2) pure PLA membrane at 5000× magnification, (b-1) pure PLA film at 750× magnification, and (b-2) pure PLA film at 5000× magnification.

**Figure 2:**
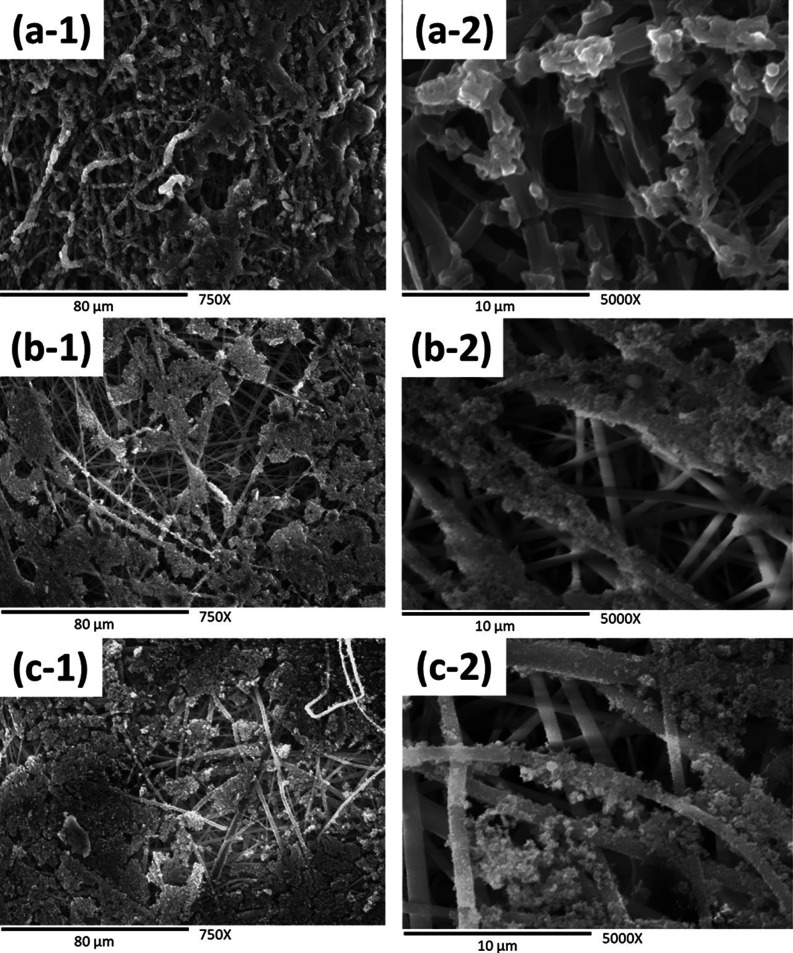
SEM micrographs of PANi-coated membranes at different polymerization times: (a-1) PLA–PANi-3 min at 750× magnification, (a-2) PLA–PANi-3 min at 5000× magnification, (b-1) PLA–PANi-5 min at 750× magnification, (b-2) PLA–PANi-5 min at 5000× magnification, (c-1) PLA–PANi-15 min at 750× magnification, and (c-2) PLA–PANi-15 min at 5000× magnification.

In this study, extruded films and electrospun membranes were fabricated by using PLA, as well as composite films and membranes composed of PLA and PANi. To thoroughly analyze the morphology of these polymeric membranes and films as well as the resulting composite materials, an extensive investigation was conducted using scanning electron microscopy (SEM). Figure 1 displays micrographs of the pure membranes and films, captured at magnifications of 750× and 5000×. For the membranes (Figure 1a-1,a-2), the images reveal the formation of randomly oriented fibers, giving them a ribbon-like morphology. Besides, these membranes exhibit a homogeneous surface devoid of particle inclusions.^33^ To determine the average diameter of the fibers, an image analysis was performed by using the ImageJ software. A line perpendicular to the orientation of each fiber was drawn, and its length was compared to the length of the scale bar present in the SEM micrograph. The results yielded an average fiber diameter of 0.40 ± 0.07 μm for PLA. On the other hand, the PLA films (Figure 1b-1,b-2) displayed a smooth and uniform surface.

Figure 2 presents micrographs illustrating the evolution of composite membranes as a function of the polymerization time. Notably, a polymerization time of 3 min (PLA–PANi-3 min), as depicted in images (a-1) and (a-2), proved to be the most suitable, as it was sufficient to achieve complete membrane coverage. In these images, a homogeneous surface with distinctive PLA fibers and the presence of solid particle aggregates corresponding to PANi can be observed.^32^ As the polymerization time was extended to 5 and 15 min, as evidenced in images (b-1), (b-2), (c-1), and (c-2), an increased agglomeration of PANi and a less uniform coating on the surface of pure PLA membranes were observed. These findings underscore the critical influence of polymerization time on the morphology and uniformity of the composite membranes. A longer polymerization time led to the formation of less homogeneous structures and the agglomeration of PANi on the membrane’s surface.

**Figure 3:**
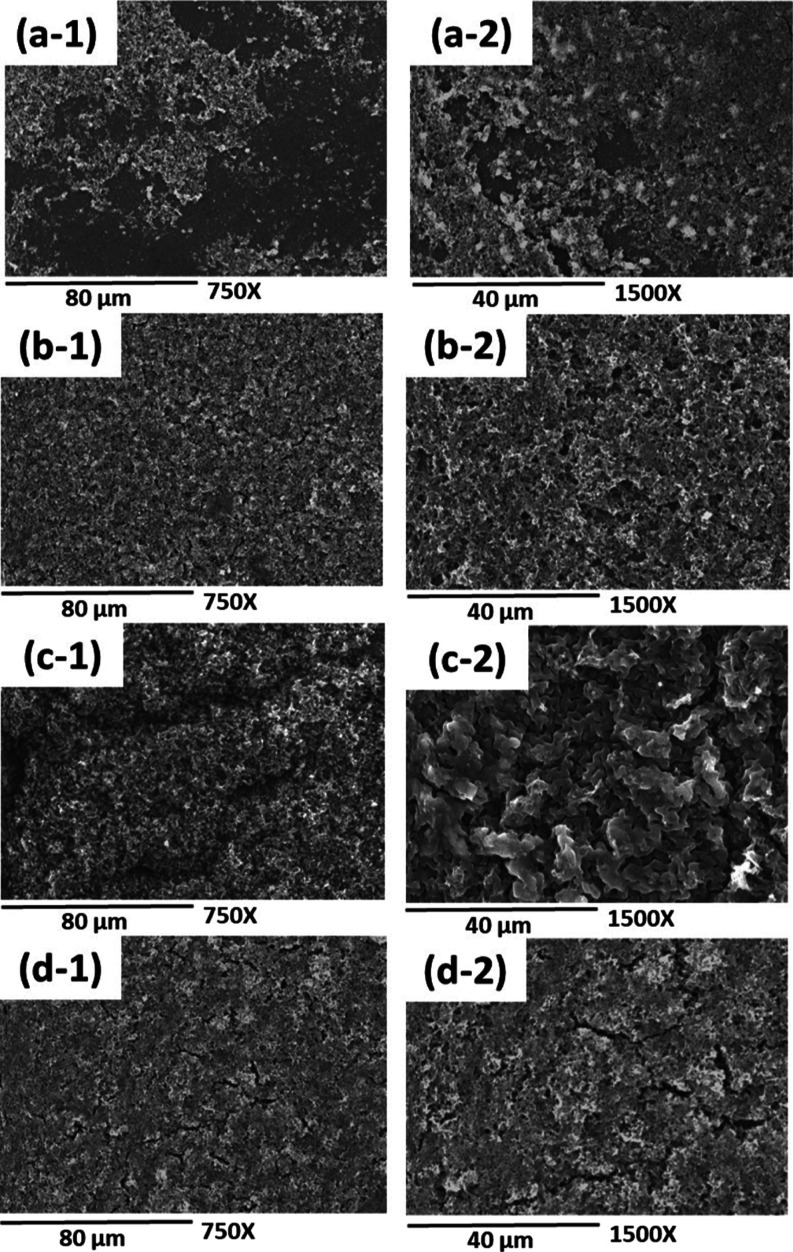
SEM micrographs of PANi-coated films at different polymerization times: (a-1) PLA–PANi-5 min at 750× magnification, (a-2) PLA–PANi-5 min at 5000× magnification, (b-1) PLA–PANi-15 min at 750× magnification, (b-2) PLA–PANi-15 min at 5000× magnification, (c-1) PLA–PANi-30 min at 750× magnification, (c-2) PLA–PANi-30 min at 5000× magnification, (d-1) PLA–PANi-1h at 750× magnification, and (d-2) PLA–PANi-1h at 5000× magnification.

**Figure 4:**
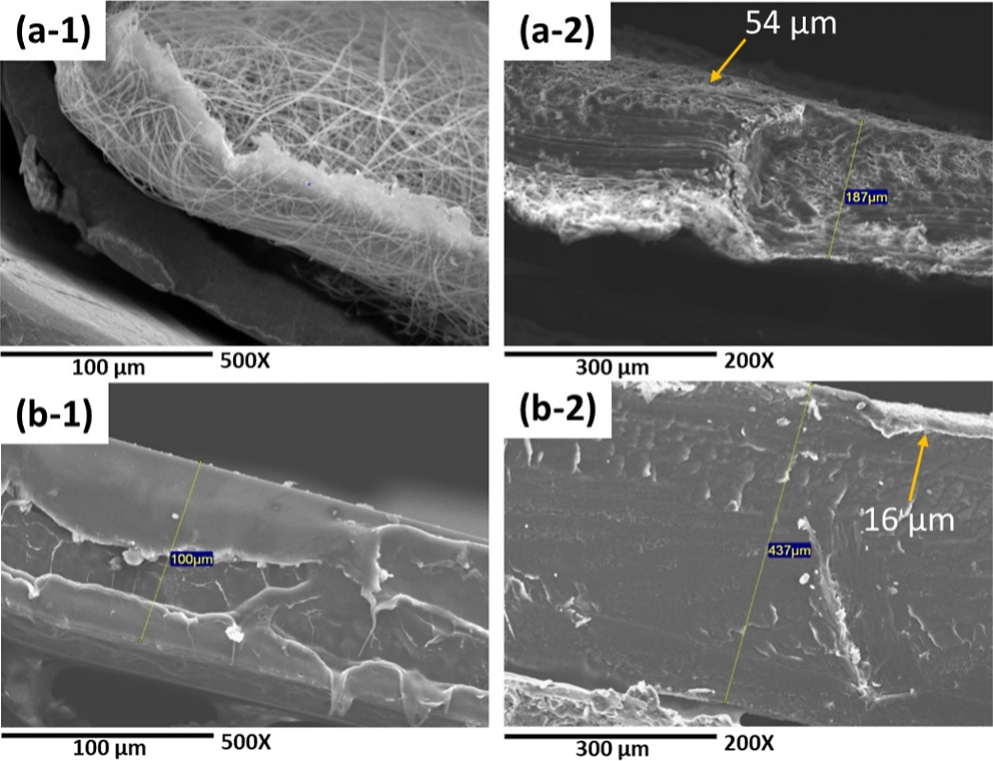
Cross-sectional micrographs of (a-1) PLA membranes at 500× magnification, (a-2) PLA–PANi-3 min at 200× magnification, (b-1) PLA films at 500× magnification, and (b-2) PLA–PANi-30 min at 200× magnification.

In Figure 3, micrographs of PLA films coated with PANi at various polymerization times are presented. Within a polymerization period of 5 and 15 min, as depicted in (a-1), (a-2) and (b-1), (b-2), respectively, the coating exhibits limited effectiveness, as PANi fails to completely cover the surface of the PLA film. This is likely attributed to the short contact time with the monomeric solution, preventing the deposition of a sufficient quantity of aniline monomers on the matrix’s surface and impeding subsequent polymerization.^34,35^ As the polymerization time is extended, a greater accumulation of PANi on the PLA film’s surface is observed, resulting in complete coverage at 30 min of polymerization [(c-1), (c-2)]. At this stage, a uniform and compact distribution of PANi particles is achieved. However, for the final polymerization period, corresponding to 1 h of reaction [(d-1), (d-2)], an agglomeration of PANi particles and a nonuniform coating with the formation of cracks on the surface of the PLA film are noticeable.^36^ Finally, Figure 4 displays micrographs of the cross-sectional views of the obtained materials, both in their pure form and in their composite composition. In images (a-2) and (b-2), a thin and uniform layer of PANi over the surface of the PLA matrix, of 54 and 16 μm for the PLA–PANi-3 min membrane and PLA–PANi-30 min film, is observed, respectively.

### 3.2 Fourier Transform Infrared Spectroscopy

**Figure 5:**
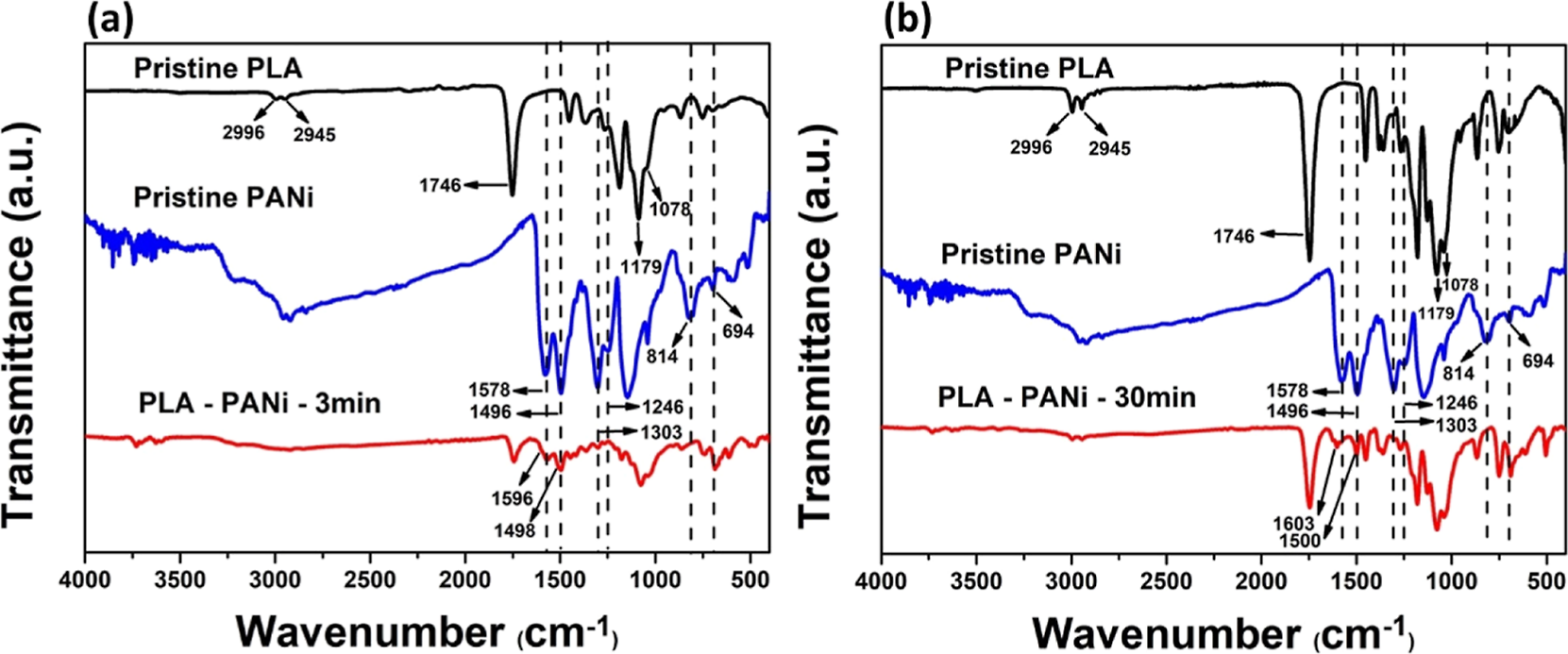
FTIR spectra of pure materials and composites: (a) PLA–PANi-3 min membrane and (b) PLA–PANi-30 min film.

Infrared spectroscopy was performed to confirm the presence or absence of characteristic functional groups in both PLA and PANi in the obtained membranes and films as well as to detect possible interactions between these groups in the composite material. In the spectra of the PLA membranes and pristine films (Figure 5), no variations were observed in the positions of the signals related to the characteristic functional groups present in these materials. This was expected, as the chemical natures of both materials are identical. The stretching band of the C=O bond appeared at a wavelength of 1746 cm^–1^, the symmetric stretches of the C–O–C bond were observed at 1078 cm^–1^, and the asymmetric stretches of the C–O–C bond were present at 1179 cm^–1^. The signals for the asymmetric and symmetric stretches of the –CH_3_ group were found at 2996 and 2945 cm^–1^, respectively. Additionally, the signals for the asymmetric and symmetric bending of this same group were detected at 1451 and 1359 cm^–1^, respectively.^37,38^ At 1266 cm^–1^, a band emerged, corresponding to the overlap of the C–H bending vibration and the stretching vibration of C–O–C.^39^ In the pristine PANi spectrum, a band at a wavelength of 1578 cm^–1^ was observed, which is attributed to the stretching of the C=N and C=C bonds present in the quinoid ring of this molecule. A signal at 1496 cm^–1^, corresponding to the stretching of the C=C bond in the benzene ring, was also identified. The stretching signals of the protonated amine C–N^+^ and the aromatic amine C–N were detected at 1303 and 1246 cm^–1^, respectively. The signals for in-plane and out-of-plane bending of the C–H bond were observed at 814 and 694 cm^–1^, respectively.^32^ The main peaks of PANi in the composite membranes PLA–PANi-3 min (Figure 5a) and in the composite films PLA–PANi-30 min (Figure 5b) were successfully identified, showing variations in the wavenumber and intensity of the peaks compared to those of pristine PANi. For the composite membranes, the peaks at 1578 and 1496 cm^–1^ shifted to 1596 and 1498 cm^–1^, respectively. For the composite films, the peaks shifted to 1603 and 1500 cm^–1^, respectively. These changes can be primarily attributed to the formation of hydrogen bond interactions between PANi and PLA.^40,41^

### 3.3 Thermogravimetric Analysis

**Figure 6:**
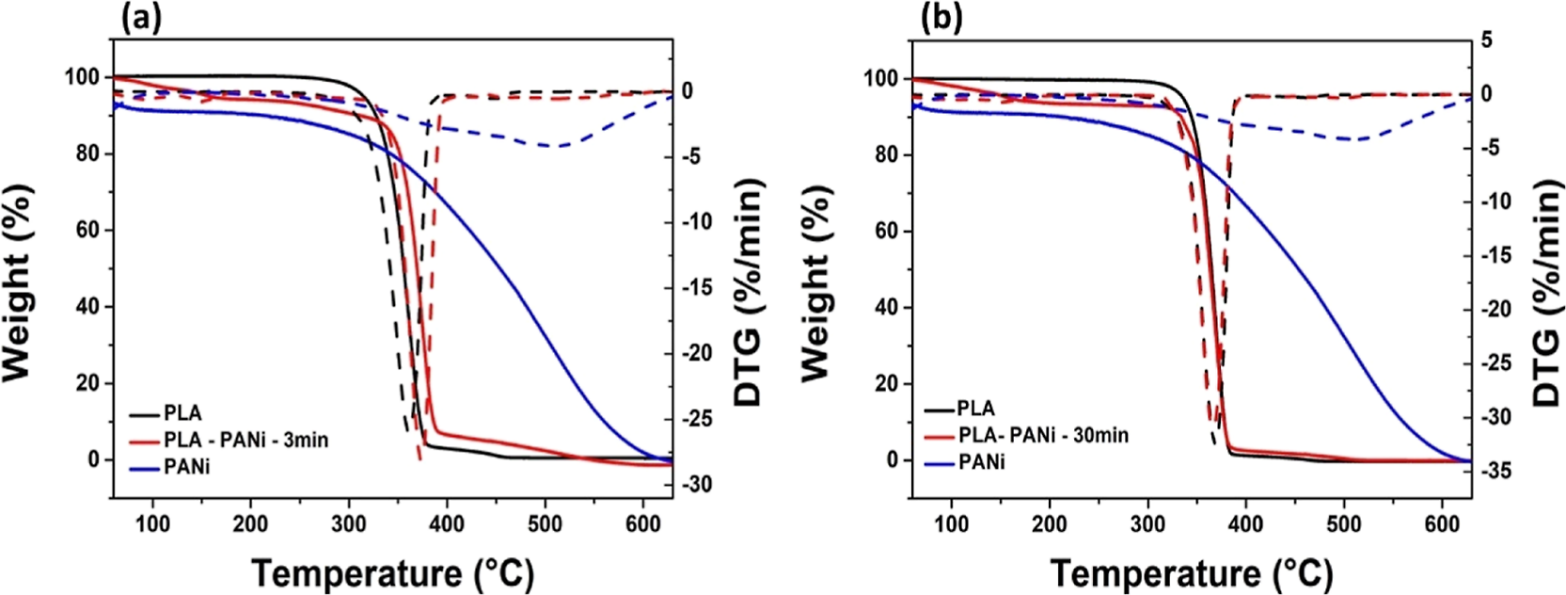
TGA and DTG curves of pure materials and composite, (a) PLA–PANi-3 min membranes and (b) PLA–PANi-30 min films.

Figure 6 displays the TGA and DTG curves for PANi and pure and composite materials. The PANi chain degraded in a single step with an initial degradation temperature (T Onset) of 264.71 °C, exhibiting a peak in temperature (Tmax) at 504.36 °C. Similarly, both the PLA membrane (Figure 6a) and film (Figure 6b) showed a single degradation peak, with initial degradation temperatures of 295.03 and 324.59 °C, respectively, corresponding to the degradation of the polyester chains. Furthermore, the PLA membrane displayed an inflection temperature of 359.95 °C, while the PLA film showed a Tmax of 367.74 °C. These results are consistent with prior literature reports.^42−44^ On the other hand, the composite membranes and films exhibited a gradual weight reduction in the temperature range of 100–200 °C, attributed to moisture loss in the materials. Furthermore, they demonstrated enhanced thermal stability compared to their pure PLA counterparts, as evidenced by their higher initial degradation temperatures of 330.52 and 325.95 °C, respectively. The inflection temperatures for these materials were 373.83 °C for the PLA–PANi-3 min membrane and 367.17 °C for the PLA–PANi-30 min film. This improved thermal stability can be attributed to intermolecular interactions between the PLA matrix and PANi particles. These findings align with the observations from the FTIR spectra (Figure 5).

### 3.4 Contact Angle

The contact angle of the PLA membrane was measured at 107.41 ± 4.35°, showing evidence of its hydrophobic nature.^45^ In contrast, the PLA film exhibited a contact angle of 76.48 ± 3.21°. This difference may be attributed to the physical heterogeneities present in membranes, such as surface roughness, which is characteristic of these materials.^46−48^ Furthermore, the contact angles of the PLA–PANi-3 min membrane and PLA–PANi-30 min film decreased to 62.63 ± 2.15 and 66.37 ± 3.87°, respectively, consistent with literature reports. This reduction can be attributed to increased hydrophilicity resulting from the presence of PANi, which in this case coats the surface of the PLA matrix.^38^

### 3.5 Tensile Strength Tests

The tensile strength, elongation at break, and Young’s modulus of the fabricated materials were calculated from the obtained stress–strain curves. Table 1 presents the obtained results. The pure PLA membrane exhibited a tensile strength of 7.37 MPa and an elastic modulus of 31.47 MPa, whereas the pure PLA film displayed a tensile strength of 40.85 MPa and an elastic modulus of 1417.97 MPa. These findings align with previous reports in the literature.^49−52^ Conversely, the incorporation of the PANi coating into the PLA matrix led to a reduction in both tensile strength and the elastic modulus of the composite PLA–PANi-30 min film, with values of 24.69 and 1283.29 MPa, respectively. These results may be attributed to the presence of a brittle polymer, such as PANi, in the composite material.^53^ Additionally, the potential formation of clusters at the interface of the PLA matrix might have induced the appearance of cracks and, consequently, material failure at lower applied stress levels.

**Table 1:** Mechanical Properties of the Processed Materials

materials	Young’s modulus (MPa)	tensile strength (MPa)	elongation at break (%)
PLA membrane	31.47 ± 6.45	7.37 ± 1.52	277.22 ± 17.82
PLA film	1417.97 ± 166.74	40.85 ± 4.88	47.41 ± 24.32
PLA–PANi-30 min	1283.29 ± 152.49	24.69 ± 4.26	4.34 ± 2.45

## 4 Conclusions

PLA membranes and films coated with PANi were successfully prepared, achieving superior coverage in the films when contact times of 30 min were employed with the monomeric solution. For the membranes, optimal results were obtained with contact times of 3 min with the monomeric solution. The evidence of the interaction between the two polymers was confirmed through thermal, spectroscopic, and contact angle characterizations. SEM characterizations revealed that the morphologies of pure films and membranes underwent significant changes after the polymerization process, indicating homogeneous deposition of PANi particles on the surface of the PLA matrix. Consequently, it can be affirmed that PLA membranes and films serve as suitable substrates for PANi. However, the composite material exhibited a decrease in its mechanical properties due to potential agglomerate formation and the inherent brittle nature of PANi. In summary, PLA membranes and films coated with PANi demonstrate potential applications in environmental remediation.
